# A Comparison of Proteins Expressed between Human and Mouse Adipose-Derived Mesenchymal Stem Cells by a Proteome Analysis through Liquid Chromatography with Tandem Mass Spectrometry

**DOI:** 10.3390/ijms19113497

**Published:** 2018-11-06

**Authors:** Saifun Nahar, Yoshiki Nakashima, Chika Miyagi-Shiohira, Takao Kinjo, Naoya Kobayashi, Issei Saitoh, Masami Watanabe, Hirofumi Noguchi, Jiro Fujita

**Affiliations:** 1 Department of Infectious, Respiratory, and Digestive Medicine, Graduate School of Medicine, University of the Ryukyus, Okinawa 903-0215, Japan; snaharmd@gmail.com (S.N.); fujita@med.u-ryukyu.ac.jp (J.F.); 2 Department of Regenerative Medicine, Graduate School of Medicine, University of the Ryukyus, Okinawa 903-0215, Japan; chika@med.u-ryukyu.ac.jp; 3 Department of Basic Laboratory Sciences, School of Health Sciences in the Faculty of Medicine, University of the Ryukyus, Okinawa 903-0215, Japan; kinjotko@med.u-ryukyu.ac.jp; 4 Okayama Saidaiji Hospital, Okayama 704-8192, Japan; n-kobayashi@saidaiji-hp.or.jp; 5 Division of Pediatric Dentistry, Graduate School of Medical and Dental Science, Niigata University, Niigata 951-8514, Japan; isaito@dent.niigata-u.ac.jp; 6 Department of Urology, Okayama University Graduate School of Medicine, Dentistry and Pharmaceutical Sciences, Okayama 700-8558, Japan; masami5@md.okayama-u.ac.jp

**Keywords:** adult stem cells, mesenchymal stem cells, regenerative medicine

## Abstract

Adipose-derived mesenchymal stem cells (ADSCs) have become a common cell source for cell transplantation therapy. Clinical studies have used ADSCs to develop treatments for tissue fibrosis, such as liver cirrhosis and pulmonary fibroma. The need to examine and compare basic research data using clinical research data derived from mice and humans is expected to increase in the future. Here, to better characterize the cells, the protein components expressed by human ADSCs used for treatment, and mouse ADSCs used for research, were comprehensively analyzed by liquid chromatography with tandem mass spectrometry. We found that 92% (401 type proteins) of the proteins expressed by ADSCs in humans and mice were consistent. When classified by the protein functions in a gene ontology analysis, the items that differed by >5% between human and mouse ADSCs were “biological adhesion, locomotion” in biological processes, “plasma membrane” in cellular components, and “antioxidant activity, molecular transducer activity” in molecular functions. Most of the listed proteins were sensitive to cell isolation processes. These results show that the proteins expressed by human and murine ADSCs showed a high degree of correlation.

## 1. Introduction

Mesenchymal stem cells (MSCs) [1] are a source of cells for cell therapy in regenerative medicine. These are currently being evaluated in clinical trials aimed at determining their spectrum of efficacy. The multiple sources of MSCs include the placenta, umbilical cord blood, peripheral blood, skeletal muscle, adult bone marrow, lungs, amniotic fluid, and adipose tissue. Among these sources, the abundance and accessibility of adipose tissue-derived MSCs (ADSCs) [2,3,4] have proven superior to those of other sources of MSCs, suggesting that these cells will be more commonly used in cellular therapy [5,6,7]. ADSCs are useful for addressing a multitude of diseases, including osteoarthritis and diabetes mellitus. These cells can differentiate into bone or muscle and help soft tissue regeneration. The implied use of ADSCs as a superior source of MSCs suggests the need for the functional characterization and quantitative studies of ADSCs. 

Basic research to elucidate the characteristics and functions of ADSCs in detail using experimental animals such as mice is extremely important for confirming the safety and efficacy of ADSC cell therapy for humans. However, it is often noted that the results of basic research using mice contradict the results of clinical studies targeting humans. Thus, experimental results from the comparison of the proteins expressed in mouse and human ADSCs would likely be useful as preliminary data when cell therapy using ADSCs is performed in humans. 

Gene Ontology (GO), developed at the GO Consortium, provides genomic sequencing and annotation tools related to the functions of genes or “GO terms”. GO describes these functions based on their activity, location, and connected processes. In the past 18 years, the GO subsets have been well established and have been widely used in various studies, including (but not limited to) studies involving the use of mice, yeast, nematodes, and meta-genomics. GO facilitates the development of a common vocabulary to describe biological concepts [8,9]. Since the late 1990s, various aspects of gene-related information have been aggregated and stored in databases thanks to the innovation of experimental methods in biology (e.g., DNA sequencers, DNA microarrays, etc.) and the development of bioinformatics methodologies. Efforts have been made to classify the functions of genes (proteins) to facilitate the organization of the gene and protein names registered in databases into mutually usable information on different databases. The terms defined in GO are called ‘GO terms’. These are divided into three categories: biological processes, cellular components, and molecular functions. In recent years, liquid chromatography with tandem mass spectrometry (LC-MS/MS) has been used to perform the GO classification of comprehensive expression data using protein analysis software programs. 

Three types of cell, tissue, and organ transplantation are performed: autotransplantation, where a part of a person is transplanted to another part of the same person; allotransplantation, where a part is transplanted from one individual to another individual of the same species; and xenotransplantation, where a part is transplanted from an individual of one species to an individual of another species. Among these types of transplantation, allotransplantation is associated with a risk of an immune rejection reaction due to a blood type mismatch of leukocytes called human leukocyte antigen (HLA). In order to solve this problem, the usefulness of therapeutic cells using cells with HLA homozygotes, which are less susceptible to immunological rejection, has been indicated. In xenotransplantation, in addition to differences in HLA, there is a risk of severe immuno-elimination as a result of differences between the species of cell surface markers. Many causes of immune rejection among heterologous cells are due to species differences of different cell surface markers. There are cases in which cell surface marker variations differ and the protein surface structure of cell surface markers differ according to species differences. As examples of the former issue, there are reports of many species differences in hADSCs (CD45−/CD31−/CD34+) [10,11,12] and mADSCs (CD45−/CD31−/Sca1+) [13,14].

In this work, we studied adipose-derived mesenchymal stem cells (ADSCs) using liquid chromatography-tandem mass spectrometry (LC-MS/MS) and categorically compared the characteristics with the available GO data on ADSC cells.

## 2. Results

### 2.1. Characteristics and Cell Qualities of hADSCs

**Figure 1:**
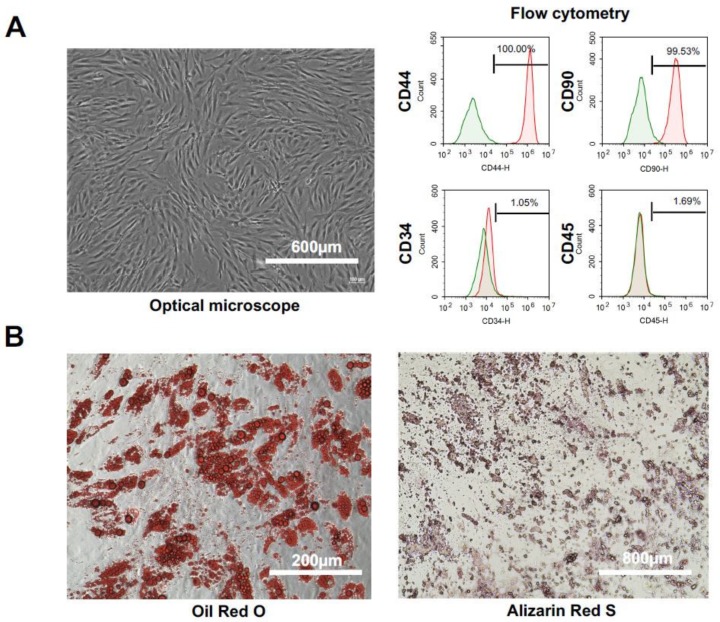

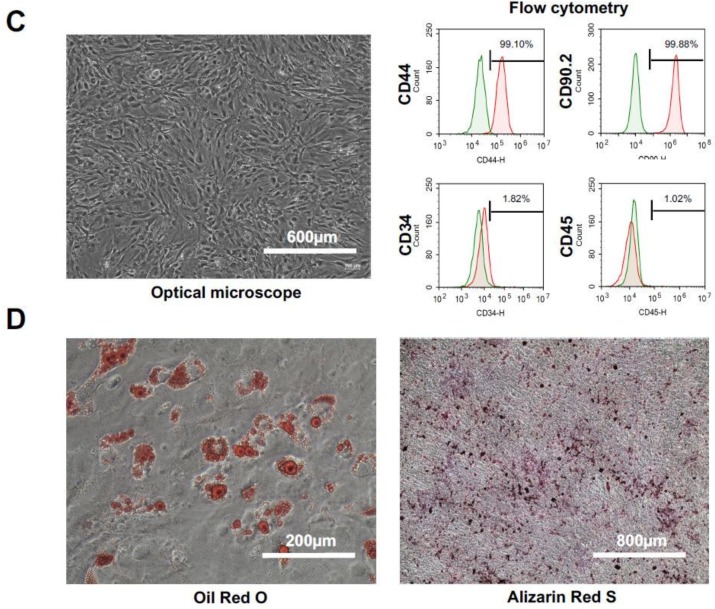
The phenotype and differentiation potential of hADSCs and mADSCs in culture. The morphological appearance of hADSCs (passage 3) (**A**), (left panel). The characterization of hADSCs (passage 3). The characterization of hADSCs was performed by flow cytometry using antibodies against CD34, CD44, CD45, and CD90. hADSCs were positive for CD44, and CD90, but negative for CD34 and CD45 (**A**), (right panels). Representative images of adipocyte (**B**), (left panel) and osteocyte differentiation (**B**), (right panel) from hADSCs (passage 3) cultured in differentiation medium. The morphological appearance of mADSCs (passage 3) (**C**), (left panel). The characterization of mADSCs (passage 3). The characterization of mADSCs was performed by flow cytometry using antibodies against CD34, CD44, CD45, and CD90.2. mADSCs were positive for CD44, and CD90.2, but negative for CD34 and CD45 (**C**), (right panels). Representative images of adipocyte (**D**), (left panel) and osteocyte differentiation (**D**), (right panel) from hADSCs (passage 3) cultured in differentiation medium.

hADSCs were cultured to 80% confluence using Dulbecco’s modified Eagle medium (DMEM) medium containing 10% fetal bovine serum (FBS). We observed no abnormalities in cell size, shape, or culture state with a normal microscope (Figure 1A, left panel). Our university conducts human clinical research using ADSCs at the Regenerative Medicine Research Center (RMRC) of the University of the Ryukyus School of Medicine. In accordance with the Japanese Ministry of Health, Labor and Welfare Ordinance No. 110 (26 September 2014), “Standard of quality inspection (cell marker) of ADSCs usable for clinical research”, the RMRC applies three evaluation criteria for ADSCs that can be administered to the body of a patient, namely: (1) the proportion of viable cells (viability > 70%); (2) sterility, as determined by mycoplasma and an endotoxin assay; and (3), the experimental results of a cell surface marker expression analysis using flow cytometry. The latter criterion mandates positivity for CD29 > 95%, CD44 > 95%, ScaI > 95% (reference: CD90 > 85% lower than others (CD29, CD44 and ScaI)); and negativity for CD34 < 5% and CD45 < 5%. Flow cytometry was performed using markers of hADSC (CD44, CD90), hematopoietic stem cells (CD34) and leukocytes (CD45). CD44 and CD90 were expressed in hADSCs, while CD34 and CD45 were not detected (Figure 1A, right panels). We induced differentiation into adipocytes (Figure 1B, left panel) and osteoblasts (Figure 1B, right panel) using hADSCs. Mature adipocytes were stained with Oil Red O, and mature osteoblasts were stained with Alizarin Red S. The results confirmed that hADSCs isolated from fat were induced to differentiate into Oil Red O staining-positive adipocytes after the induction of adipocyte differentiation, and that hADSCs were induced to differentiate into Alizarin Red S-positive osteoblasts after the induction of osteoblastic differentiation. These results show that hADSCs isolated from fat are mesenchymal stem cells with the ability to differentiate into fat and bone.

### 2.2. Characteristics and Cell Qualities of mADSCs

mADSCs were cultured to 80% confluence using DMEM medium containing 10% FBS. We observed no abnormalities in cell size, shape, or culture state with a normal microscope (Figure 1C, left panel). Flow cytometry was performed using markers of mADSCs (CD44, CD90.2), hematopoietic stem cells (CD 34) and leukocytes (CD 45). CD44 and CD90.2 were expressed in mADSCs while CD34 and CD45 were not detected (Figure 1C, right panels). We induced differentiation into adipocytes (Figure 1D, left panel) and osteoblasts (Figure 1D, right panel) using mADSCs. Mature adipocytes were stained with Oil Red O, and mature osteoblasts were stained with Alizarin Red S. The results confirmed that mADSCs isolated from fat were induced to differentiate into Oil Red O staining-positive adipocytes after the induction of adipocyte differentiation, and that mADSCs were induced to differentiate into Alizarin Red S-positive osteoblasts after the induction of osteoblastic differentiation. These results show that mADSCs isolated from fats are mesenchymal stem cells with the ability to differentiate into fat and bone.

### 2.3. Comprehensive Protein Expression Analysis of hADSCs and mADSCs

**Figure 2:**
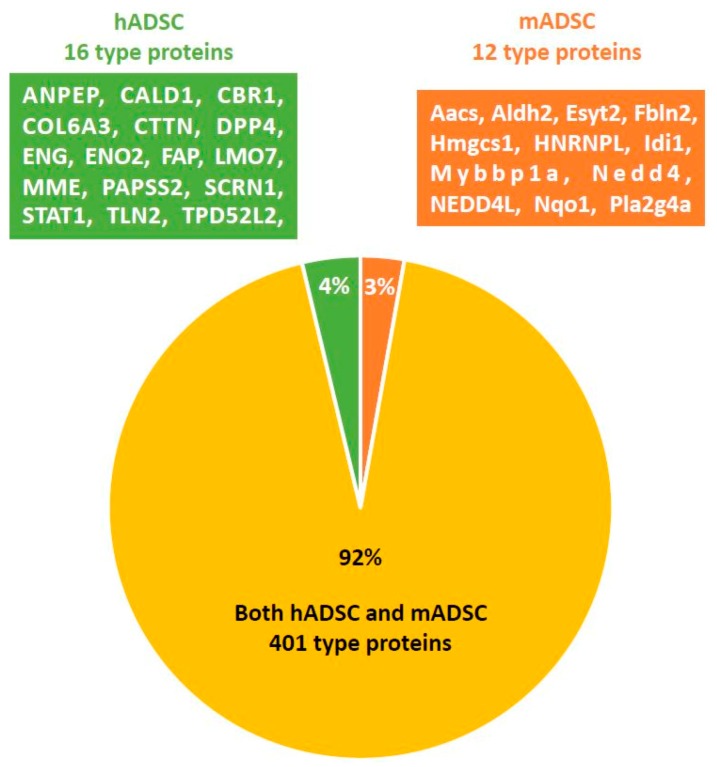
A Venn diagram of proteins detected by LC-MS/MS. A total of 417 proteins were identified from cultured hADSCs (Passage(P)3) and 413 proteins were identified from cultured mADSCs (P3). Among the 417 proteins identified in hADSCs, 16 were unique to hADSCs (hADSC group). Among the 413 proteins identified in mADSCs, 12 were unique to mADSCs (mADSC group). A total of 401 proteins were identified in both hADSCs and mADSCs (both hADSC and mADSC group).

A total of 417 proteins were identified from hADSC samples and 413 proteins were identified from mADSC samples. Of the 417 proteins identified from hADSCs, 16 (4%) were unique to hADSCs (ANPEP, CALD1, CBR1, COL6A3, CTTN (Src substrate cortactin), DPP4 (Dipeptidyl peptidase 4), ENG (Endoglin), ENO2, FAP (Prolyl endopeptidase FAP ((also known as fibroblast activation protein)), LMO7, MME, PAPSS2, SCRN1, STAT1, TLN2, TPD52L2). A total of 401 proteins in mADSCs were also identified in hADSCs (both hADSCs and mADSCs). Of the 413 proteins identified from mADSCs, 12 (3%) were specific to mADSCs (Aacs, Aldh2, Esyt2, Fbln2, Hmgcs1, HNRNPL, Idi1, Mybbp1a, Nedd4, NEDD4L, Nqo1, Pla2g4a). Thus, the highly expressed proteins of hADSCs and mADSCs were 92% identical (Figure 2).

### 2.4. GO Classification of Proteins Expressed in hADSCs and mADSCs

**Figure 3:**
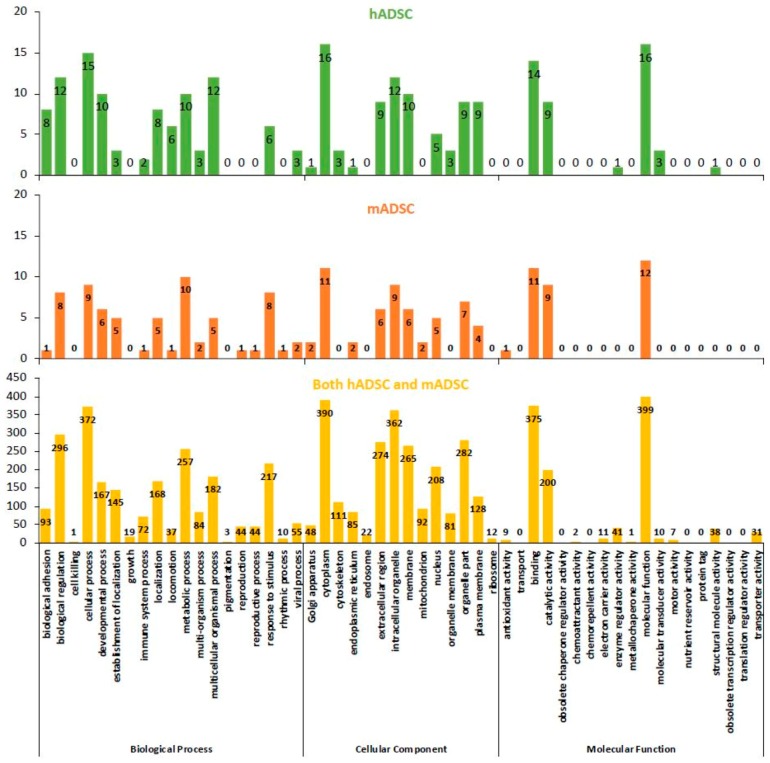
The biological processes, cellular components, and molecular functions of the hADSC, mADSC, and both hADSC and mADSC groups (determined by the Gene Ontology, GO, analysis). The principal component analysis (PCA) of proteome dynamics based on the protein information generated by high-resolution mass spectrometry. The abscissa indicates the biological function, cellular component, and molecular function of the protein. The ordinate indicates the number of proteins identified in the hADSC (top panel), mADSC (middle panel) and both hADSC and mADSC groups (bottom panel). The names of the proteins classified in Table 1 and Supplementary Table S1 are listed according to their detailed biological process (biological adhesion, locomotion, and rhythmic process), cellular component (plasma membrane), and molecular function (antioxidant activity and molecular transducer activity).

The Gene Ontology Consortium (http://www.geneontology.org/) is a database of functional information aimed at describing biological phenomena in standardized terms. In addition, it is designed to capture biological phenomena exhaustively regardless of the species, with GO terms largely represented by three subcategories: biological processes, cellular components, and molecular functions. The proteins uniquely expressed in hADSCs were classified by a GO analysis (hADSC group; Figure 3, top panel), as were those that were uniquely expressed in mADSCs (mADSC group; Figure 3, middle panel) and in both hADSCs and mADSCs (both hADSC and mADSC group; Figure 3, lower panel). The biological process, cellular component and molecular function classifications are shown on the left, middle, and right of Figure 3, respectively. The results of the GO analysis indicated that the classifications of the proteins expressed by hADSCs and mADSCs were similar.

### 2.5. Comparison of the Rates of the GO Findings

**Figure 4:**
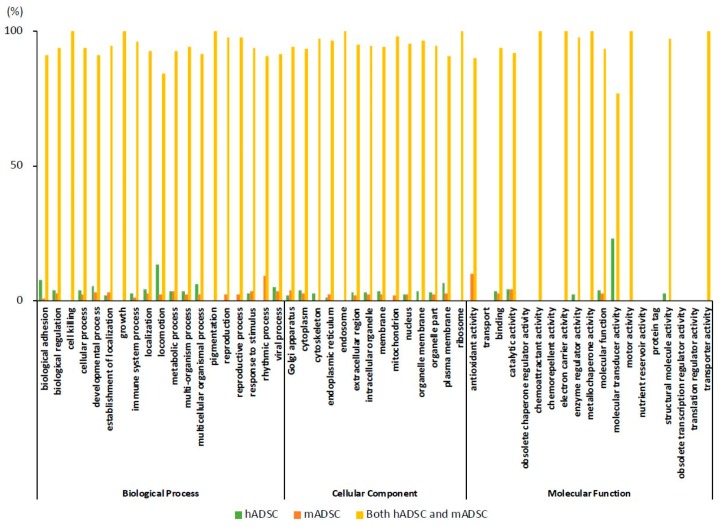
The GO composition ratio of the number of proteins in the hADSC, mADSC, and both hADSC and mADSC groups. The values calculated by dividing the number of proteins in the GO-classified hADSC, mADSC, both hADSC and mADSC groups by the total number (hADSC + mADSC + both hADSC and mADSC) are displayed (%). The protein counts in the hADSC, mADSC, and both hADSC and mADSC groups in Figure 3 are shown after the percentage was calculated. Supplementary Figure S2 shows the figure with the maximum value on the Y-axis of Figure 4 set at 10%.

We expressed the GO classifications as percentages (Figure 4). When classified by the protein function in the GO analysis, the items that showed >5% difference between hADSCs and mADSCs were: “biological adhesion” (hADSC 7.8%; mADSC 1.0%), “locomotion” (hADSC 13.6%; mADSC 2.3%), and “rhythmic process” (hADSC 0.0%; mADSC 9.1%) in biological processes; “plasma membrane” (hADSC 6.4%; mADSC 2.8%) in cellular components; and, “antioxidant activity” (hADSC 0.0%; mADSC 10.0%) and “molecular transducer activity” (hADSC 23.1%; mADSC 0.0%) in molecular functions. All of the other GO classifications of hADSCs and mADSCs showed a > 95% match. All of the protein names corresponding to the GO classifications for which a > 5% mismatch existed among various protein functions classified in the GO analysis are shown in Supplementary Table S1.

## 3. Discussion

This work presents a simple yet comprehensive qualitative and quantitative study of the functional characteristics of ADSCs. We compared human and mouse ADSCs. We found that 92% (401 type proteins) of the proteins expressed by ADSCs in humans and mice were consistent. Recently, in a comprehensive protein expression analysis using the iTRAQ measurement method [15], in which peptide fragments were tagged before performing an analysis similar to LC-MS/MS, it was reported that more than 2000 types of proteins were identified from hADSCs [16]. In this study, 1653 proteins with one or more peptide fragments was identified in hADSCs, and 1623 were identified in mADSCs. The number of species detected by these proteins (emPAI value > 0) is considered to be close to the number of species detected using iTRAQ. However, when the number of samples is small, we believe that it is not appropriate to conclude that a protein is expressed based on the presence of one peptide fragment. As the emPAI value increases, the number of peptide fragments detected increases. According to a previous study, we reported that an emPAI value of >10 is a reliable protein detection condition when a single sample is analyzed [17]. Therefore, only proteins with an emPAI value of >10, which were detected at high frequency by an exhaustive protein expression analysis, were included in the present study. Thus, the number of protein species detected was ≤500.

In this study, mADSCs were isolated from adipose tissue by almost the same protocol that is used to isolate hADSCs for clinical use. Then, using a medium composed of the same ingredients, cells that were passaged three times were used as samples for a comprehensive protein expression analysis. However, because of the small amount of adipose tissue in mice, the number of cell divisions in both hADSCs and mADSCs, even when the number of passages is the same. Indeed, it is well known that the culturing and passaging of adipose progenitors will lead to homogenization [18]. Recently, we performed a comprehensive protein expression analysis using mADSCs on passages zero and three. Furthermore, it was reported that the component protein of the stromal vascular fraction (SVF) was contained in a passage zero group [19]. Generally, in the clinical setting, it is recommended that hADSCs be passaged three to five times. Thus, in the analysis of this study, we used three passages, which matches the conditions under which hADSCs are used in the clinical setting.

A notable finding of this study is that hADSCs expressed eight proteins (TLN2, COL6A3, CALD1, FAP, STAT1, ENG, DPP4, and CTTN) related to cell adhesion, which was more than mADSCs (one protein related to cell adhesion, Esyt2). According to previous studies, the function of TLN2 expressed in hADSCs is classified as “migration and angiogenesis promoting”. It was reported that the expression of TLN2 in hADSCs was increased by the administration of IL-2 [20]. Previous studies reported that COL6A3 mRNA is highly expressed by small adipocytes [21]. Another paper reported a correlation between the mRNA expression levels of fibrotic protein COL5A1 and COL6A3 expressed in hADSCs [22]. It was previously reported that CALD1, a cytoskeleton, was not expressed in human fibroblasts but was expressed in hADSCs [23]. Previously, FAP was reported to be an essential factor for cell migration associated with RhoA activity and was specific to human bone marrow-derived mesenchymal stem cells [24]. Our detection of FAP protein fragments in hADSCs in the present experiment suggested that the same mechanism may be present in hADSCs. Previous reports showed that STAT1 signaling is involved in interferon (IFN)-γ-induced immunosuppression in human bone marrow-derived mesenchymal stem cells [25]. In the present study, a STAT1 protein fragment was detected in hADSCs, suggesting that a similar mechanism may exist in hADSCs. Previously, endoglin (ENG, also known as CD105) was reported as an hADSC-specific cell surface marker [26]. The membrane glycoprotein dipeptidyl peptidase-4 (also known as DPP4, CD26) [27] is widely expressed, especially in endothelial cell, epithelial cell and immune cell populations. However, no study has reported that DPP4 is expressed in hADSCs. Thus, careful judgment is required to determine whether the protein fragment of DPP4 detected in hADSCs by our measurement is derived from hADSCs or some other contaminating cells. CTTN contributes to the organization of the actin cytoskeleton and cell shape. However, the expression of CTTN in hADSCs has not been reported. Using the Human Protein Atlas (https://www.proteinatlas.org/ENSG00000085733-CTTN/tissue), it can be confirmed that in addition to being expressed in adipose tissue, CTTN mRNA and protein are expressed in various organs of the body.

In addition, hADSCs expressed six proteins (FAP, STAT1, ENG, DPP4, CTHRC 1, and CTTN) related to locomotion, which was more than mADSCs (one protein related to locomotion, Nedd4). mADSCs also expressed one protein related to the rhythmic process (Mybbp1a). hADSCs expressed nine proteins classified as cellular components in the plasma membrane (TLN2, COL6A3, CALD1, ANPEP, FAP, MME, ENG, DPP4, and CTTN), while mADSCs expressed four such proteins (Hmgcs1, Esyt2, Nedd4, and NEDD4L). These results show that 92% of the highly expressed proteins corresponded between hADSCs and mADSCs, and a GO analysis of the 8% of mismatched proteins primarily turned up cell surface proteins involved in cell adhesion. The cell membrane proteins and cell adhesion-related proteins might have been affected by the methods that were used to isolate and separate hADSCs and mADSCs. 

The hADSCs and mADSCs express macrophage migration inhibitory factor (MIF) protein (Supplementary Table S1). MIF is a pleiotropic inflammatory cytokine involved in various inflammatory diseases. It has been reported that MIF knockout mice showed a strong increase in fibrosis in a mouse model of chronic liver injury. MIF has an anti-fibrotic effect on the liver via the MIF receptor (CD74). In addition, recombinant MIF proteins have similar anti-fibrotic effects [28]. These results indicate that MIF secreted by ADSCs is a major component in the control of liver fibrosis.

In this study, mADSCs were prepared in the same way as hADSCs are prepared for human clinical studies, and the two cell types were cultured under the same conditions. As a result, the patterns of proteins expressed by hADSCs and mADSCs were highly similar. This result therefore provides a rationale for the utilization of basic research data obtained using hADSC (animal experiments using mice) before researchers and doctors work on human clinical research using hADSCs. Nevertheless, the data from the present study cannot be used to predict whether the protein components expressed in MSCs derived from mice or other tissues taken from human beings will be similar, and additional research is necessary.

A recent paper reported that symptoms in a mouse model of acute liver failure model were improved using a secretory protein of hADSCs [29]. In this regard, we expect that a comparison of proteins secreted by hADSCs and mADSCs would prove interesting. Importantly, we are not yet able to determine the difference between the administration of ADSCs and the injection of ADSC-secreted proteins as treatment in the clinical setting; thus ADSCs were administered in the present study.

## 4. Materials and Methods

### 4.1. Reagents

Fetal bovine serum (FBS) was obtained from BioWest (Nuaille, Maine-et-Loire, France). Dulbecco’s modified Eagle medium (DMEM) [D-MEM (High Glucose) with L-glutamine, phenol red and sodium pyruvate (catalogue number 043-30085)] was obtained from Wako Pure Chemical Industries (Osaka, Japan). hADSCs (46-year-old Caucasian female) were purchased from PromoCell (Heidelberg, Germany). The plastic dishes were obtained from TPP (Trasadingen, Switzerland).

### 4.2. Animal Care

All experimental protocols were performed according to the guidelines for the care and use of laboratory animals set by the Research Laboratory Center, Faculty of Medicine, and the Institute of Animal Experiments, Faculty of Medicine, University of the Ryukyus (Okinawa, Japan). The experimental protocol was approved by the Committee on Animal Experiments of University of the Ryukyus (permit number: A2017101). C57BL/6 male mice (8 weeks of age; Japan SLC, Shizuoka, Japan) were housed in a controlled environment (23 ± 2 °C temperature, lights on from 8:30 a.m. to 8:30 p.m.). Animals were fed standard rodent chew pellets with *ad libitum* access to water. All efforts were made to minimize the suffering of the animals.

### 4.3. Isolation of Mouse ADSCs from Adipose Tissue

Adipose tissue specimens (ATs) were collected from the inguinal fat pad of 8-week-old C57BL/6 mice. The method of isolating MSC-AT from adipose tissue was in accordance with the ADSCs product standard document (RMRC-A 01: 2015) of the Ryukyus Regenerative Medicine Research Center. In brief, ATs were stored in cold Hanks’ balanced salt solution (HBSS) after collection and washed three times using HBSS before starting the digestion procedure. The ATs were then minced using scissors and tweezers to enhance enzymatic digestion. Digestion was performed in 2 mg/mL collagenase type IV (Sigma Aldrich, St. Louis, MO, USA) and HBSS in a 50-mL centrifuge tube at 37 °C in a shaker (BioShaker BR-42FM; TAITEC, Saitama, Japan) at 20 rpm with manual shaking at 10-min intervals. After completing digestion, centrifugation was performed at 800× *g* for 5 min followed by the removal of the upper fibrous layer and supernatant. The cell pellet was suspended in fresh DMEM containing 10% FBS to inhibit collagenase activity, followed by filtration through a 100-μm cell strainer. Filtered cells were then incubated in a T25 flask using DMEM containing 10% FBS. All mouse studies were approved by the Institutional Animal Care and Use Committee of the University of the Ryukyus.

### 4.4. Cell Preparation and Quality Control

#### 4.4.1. mADSCs

We performed three protein analyses of mADSCs and used their representative data in this experiment. The mADSCs were cryopreserved at passage 2, then thawed again and used for the experiments. mADSCs were cultured in a non-coated T25 flask (TPP 90026), with the appropriate culture conditions maintained (37 °C, 5% CO_2_). The medium was completely exchanged every two days. The passage of cells was performed every 3 to 4 days after reaching 80% confluence. After reaching 80–90% confluence, the cells were washed with Dulbecco’s phosphate-buffered saline (without magnesium, calcium; FUJIFILM Wako Pure Chemical Corporation, Osaka, Japan), then the adhered cells were dissociated using 0.25% trypsin and 1 mM EDTA (catalogue number CC-3232, Lonza, Walkerville, MD, USA). Subculturing was carried out in a non-coated T25 flask using DMEM containing 10% FBS. 

Mouse ADSCs (passage 3) were used for a cell quality inspection and LC-MS/MS. The quality of the cells was assessed using fluorescein-coupled antibodies against CD90, CD34, CD44, and CD45 with a Novocyte^®^ flow cytometer (ACEA Biosciences, Inc., San Diego, CA, USA). In brief, ADSCs (1 × 10^5^ cells) were mixed with 0.5 mL of Perfusion Solution (CORNING, Manassas, VA, USA). Each antibody (1/100 of the volume) was added to the cell mixture, which was then incubated on ice for 30 min. After washing the cells with Brilliant Stain Buffer (BD Biosciences, Franklin Lakes, NJ, USA), Fluorescence activated cell sorting (FACS) was performed. The following primary antibodies were used: Brilliant Violet 421TM Rat Anti-Mouse CD44 (BD Biosciences), Fluorescein Isothiocyanate (FITC) Rat Anti-Mouse CD90.2 (BD Biosciences), PerCP/Cy5.5 Anti-Mouse CD34 (Biolegend, San Diego, CA, USA), and PE/Cy7 Rat Anti-Mouse CD45 (BD Biosciences). Isotype-identical antibodies were used as controls.

#### 4.4.2. hADSCs

We regularly produce clinical hADSCs at our university. However, when patient cells are used in a protein expression analysis, the patient’s age, gender, and disease background are expected to influence the cell state. Thus, in this study, we used quality assured hADSCs that are commercially available as research hADSCs. The hADSCs were cryopreserved at passage 2, then thawed again and used for experiments. Commercially available hADSCs were cultured using DMEM containing 10% FBS under appropriate culture conditions. The medium was completely exchanged every two days. The passaging of cells was performed every 3 to 4 days after reaching 80% confluence. After reaching 80–90% confluence, the cells were washed with Dulbecco’s phosphate-buffered saline (without magnesium, calcium; Wako Pure Chemical Industries), then the adhered cells were dissociated using 0.25% trypsin and 1 mM EDTA (catalogue number CC-3232, Lonza, Walkerville, MD, USA). Passage three hADSCs were used for a cell quality inspection and LC-MS/MS. The quality of the cells was assessed using fluorescein-coupled antibodies against CD90, CD34, CD44, and CD45 with a Novocyte^®^ flow cytometer (ACEA Biosciences, Inc.). In brief, ADSCs (1 × 10^5^ cells) were mixed with 0.5 mL of Perfusion Solution (CORNING, Manassas, VA, USA). Each antibody (1/100 of the volume) was added to the cell mixture, which was then incubated on ice for 30 min. After washing the cells with Brilliant Stain Buffer (BD Biosciences), FACS was performed. The following primary antibodies were used: BV421 Mouse Anti-Human CD44, FITC anti-human CD90 (Thy1) Antibody, PerCP anti-human CD34 Antibody, PE/Cy7 anti-human CD45 Antibody (BioLegend, Inc.). Isotype-identical antibodies were used as controls.

### 4.5. Cell Proliferation

The cells were seeded onto 24-well plates at a density of 5.0 × 10^4^ cells/well. On days 3 and 5, the cells were counted using a Luna™ Automated Hemocytometer (Logos Biosystem, Annandale, VA, USA). The relative cell proliferation rate was calculated by converting the number of cells on the day of cell seeding to 100%.

### 4.6. Cell Differentiation

Adipogenic differentiation was performed using OriCell^TM^ Mesenchymal Stem Cell Adipogenic Differentiation Medium (GUXMX-90031; Cyagen Biosciences, Santa Clara, CA, USA) and a Lipid Assay Kit (AK09F; Cosmo Bio Co., Ltd., Tokyo, Japan) according to the manufacturer’s instructions. Osteogenic differentiation was performed using OriCell™ Mesenchymal Stem Cell Osteogenic Differentiation Medium (GUXMX-90021, Cyagen Biosciences) and a Calcified Nodule Staining Kit (AK21, Cosmo Bio Co., Ltd.) according to the manufacturer’s instructions.

### 4.7. Immunofluorescence Staining

Immunofluorescence staining was performed using a method introduced in our previous paper [30]. The hADSC and mADSC antibodies were the same as those used in the flow cytometry experiments. DAPI staining was used in combination in the experiments with CD90 and CD90.2.

### 4.8. Protein Identification by Nano-LC-MS/MS

Both mADSCs and hADSCs were pelleted at 3 × 10^6^ cells each after culturing, and then 300 μL of EzRIPA Lysis solution was added to the cell pellet. A protein solution of 3105 μg/mL was obtained from the mADSCs. A protein solution (3800 μg/mL) was obtained from hADSCs, and 6.0 μg of protein was used for sample preparation. Finally, 0.4 μg of protein was used for nanoLC-MS/MS. Whole-cell protein extraction was performed using an EzRIPA Lysis kit in accordance with the manufacturer’s instructions (ATTO, Tokyo, Japan). The comprehensive expression analysis of proteins using LC-MS/MS was performed according to a previously reported method [17,31]. In this study, the data obtained by LC-MS/MS were quantified by the theoretical value (emPAI) [32,33,34,35] estimated based on the function of the Scaffold software program. The ratio of the number of measured peptides showed a linear relationship with the logarithm of the protein concentration, and the number obtained by subtracting 1 from the index of the peptide number ratio was defined as the emPAI value. The larger the emPAI value, the greater the amount of protein. The numerical emPAI value is shown on the right of Table 1 and Supplementary Table S1. The data for this study used ADSCs from one mouse and one human as the source for the protein analysis. The reliability of the data should therefore be considered. To maximize reliability in our study, we performed a protein analysis focusing on the proteins detected at high concentrations with an emPAI value of 10.

### 4.9. Data Analyses

#### 4.9.1. Database Searching

Tandem mass spectrometry was performed using the Proteome Discoverer software program (version 1.4, Thermo Fisher Scientific, Tokyo, Japan). Charge state deconvolution and deisotoping were not performed. All MS/MS samples were analyzed using the Mascot software program (version 2.5.1; Matrix Science, London, UK). Mascot was set up to search the SwissProt_2017_12 database (unknown version, 555,100 entries) using a protein sample that had been degraded to a peptide sequence by digestive enzyme trypsin. Mascot was searched with a fragment ion mass tolerance of 0.60 Da and a parent ion tolerance of 5.0 PPM. Deamidation of asparagine and glutamine, oxidation of methionine, and carbamidomethylation of cysteine were specified as variable modifications in Mascot.

#### 4.9.2. Criteria for Protein Identification

The Scaffold software program (version 4.8.4; Proteome Software Inc., Portland, OR, USA) was used to validate the MS/MS-based peptide and protein identifications. Peptide identifications were accepted if the Scaffold Local FDR algorithm established that the probability of achieving an FDR of <1.0% was 95.0%. Protein identifications were accepted if the probability was >96.0% and they contained at least 1 identified peptide. Protein probabilities were assigned by the Protein Prophet algorithm. Proteins that contained similar peptides and which could not be differentiated based on an MS/MS analysis alone were grouped to satisfy the principles of parsimony. Proteins sharing significant peptide evidence were grouped into clusters.

#### 4.9.3. The Protein GO Analysis

The protein GO analysis was performed using the GO analysis function of the Scaffold 4 software program (Proteome Software, Inc., Portland, OR, USA) (http://www.proteomesoftware.com/products/scaffold/) with imported data (goa_uniprot_all.gaf (downloaded 14 October 2016)) [8] from the external GO Annotation Source database.

## 5. Conclusions

Proteins expressed in hADSCs and mADSCs were subjected to a comprehensive protein expression analysis using LC-MS/MS. GO classification was also performed using proteins with an emPAI value of 10 (high-concentration proteins). As a result, 92% of the expressed proteins were found in both hADSCs and mADSCs. Furthermore, most of the 8% mismatched proteins identified by the GO analysis were related to cell adhesion on the cell surface. The above results show that the types and functions of the proteins that were highly expressed in hADSC and mADSC are similar.

## Figures and Tables

**Table 1:** Identification of endogenous proteins contained in hADSC; mADSC.

UniProt/SWISS-		Biological Process			Cellular Component	Molecular Function		emPAI ^a^	
PROT ID	Alternate ID	Biological Adhesion	Locomotion	Rhythmic Process	Plasma Membrane	Antioxidant Activity	Molecular Transducer Activity	hMSC_P3_D	mouse_D
hADSC	TLN2	cell adhesion			plasma membrane			10.426	0
	COL6A3	cell adhesion			sarcolemma			77.722	0
	CALD1	cell-cell adhesion			plasma membrane			51.183	0
	ANPEP				external side of plasma membrane, integral component of plasma membrane		receptor activity, virus receptor activity	39.809	0
	LMO7							36.966	0
	FAP	cell adhesion	endothelial cell migration		invadopodium membrane, lamellipodium membrane, plasma membrane, ruffle membrane			35.07	0
	STAT1	cell-cell adhesion	endothelial cell migration					28.435	0
	MME				integral component of plasma membrane			26.539	0
	CBR1							16.113	0
	ENG	cell adhesion	cell chemotaxis, cell migration involved in endocardial cushion formation		external side of plasma membrane, transforming growth factor beta receptor complex		transforming growth factor beta-activated receptor activity, transmembrane signaling receptor activity	14.218	0
	TPD52L2							12.322	0
	DPP4	cell adhesion	endothelial cell migration		apical plasma membrane, invadopodium membrane, lamellipodium membrane		virus receptor activity	15.165	0
	PAPSS2							13.27	0
	CTHRC1		cell migration					12.322	0
	SCRN1							12.322	0
	CTTN	cell-cell adhesion, focal adhesion assembly	cell motility, substrate-dependent cell migration, cell extension		plasma membrane, voltage-gated potassium channel complex			10.426	0
mADSC	Mybbp1a			circadian regulation of gene expression				0	29.631
	Hmgcs1				plasma membrane			0	27.514
	Aldh2							0	22.223
	Esyt2	cell-cell adhesion			extrinsic component of cytoplasmic side of plasma membrane, integral component of plasma membrane			0	15.874
	Nedd4		transmission of virus		plasma membrane			0	33.864
	Pla2g4a							0	21.165
	Fbln2							0	17.99
	Idi1							0	16.932
	HNRNPL							0	17.99
	Aacs							0	14.815
	Nqo1					superoxide dismutase activity		0	13.757
	NEDD4L				plasma membrane			0	14.815

^a^ Exponentially Modified Protein Abundance Index (http://www.matrixscience.com/help/quant_empai_help.html).

## Supplementary Materials

The following are available online at http://www.mdpi.com/1422-0067/19/11/3497/s1.
