# Supplementary material for: A Comparison of Proteins Expressed between Human and Mouse Adipose-Derived Mesenchymal Stem Cells by a Proteome Analysis through Liquid Chromatography with Tandem Mass Spectrometry
Source: Int J Mol Sci. 2018 Nov 6;19(11):3497. doi: 10.3390/ijms19113497 (PMC6274862; doi:10.3390/ijms19113497)
Supplement: Supplementary file 1 [file ijms-19-03497-s001.pdf]

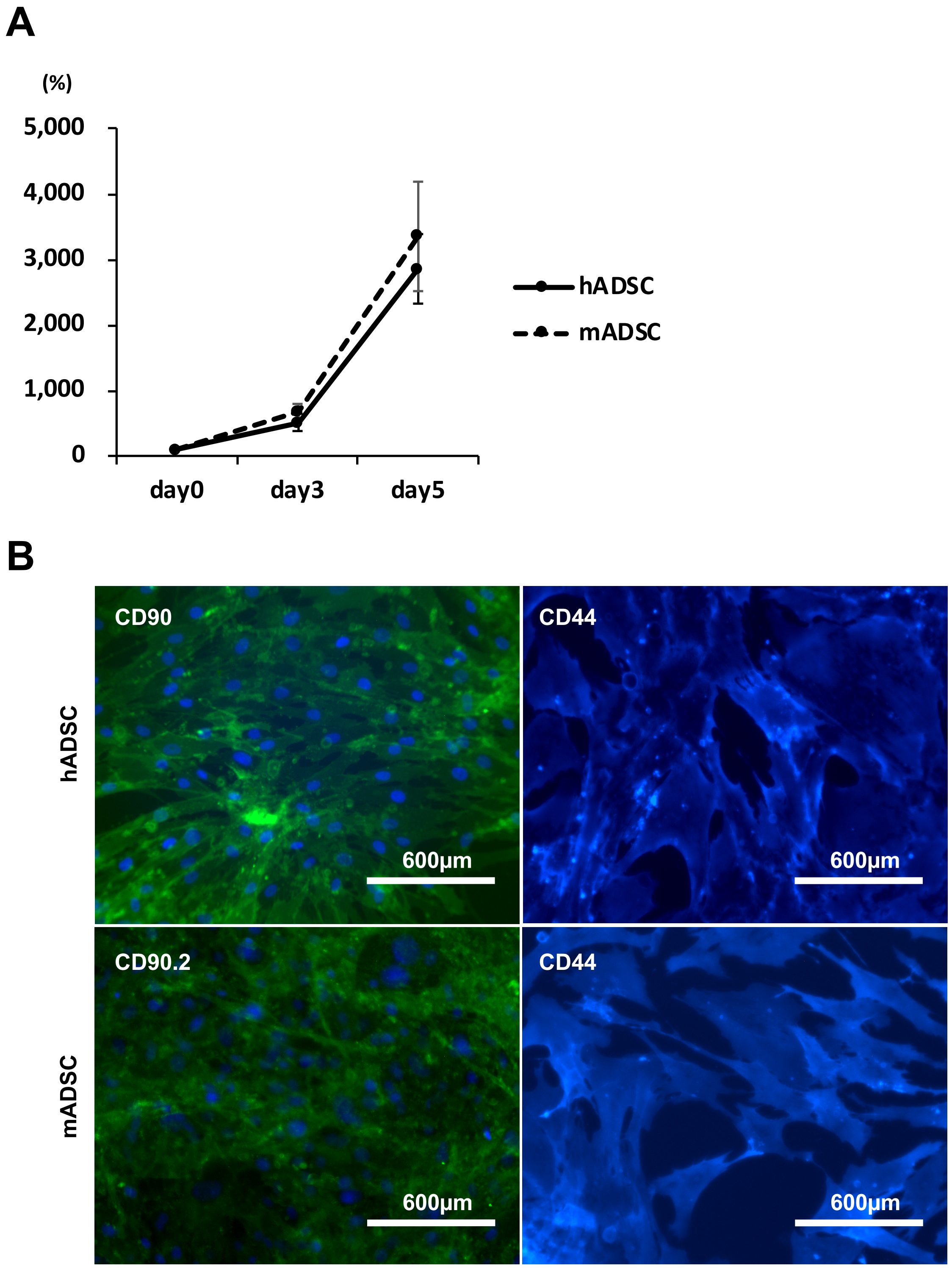

Nahar et al. Supplementary Figure 1

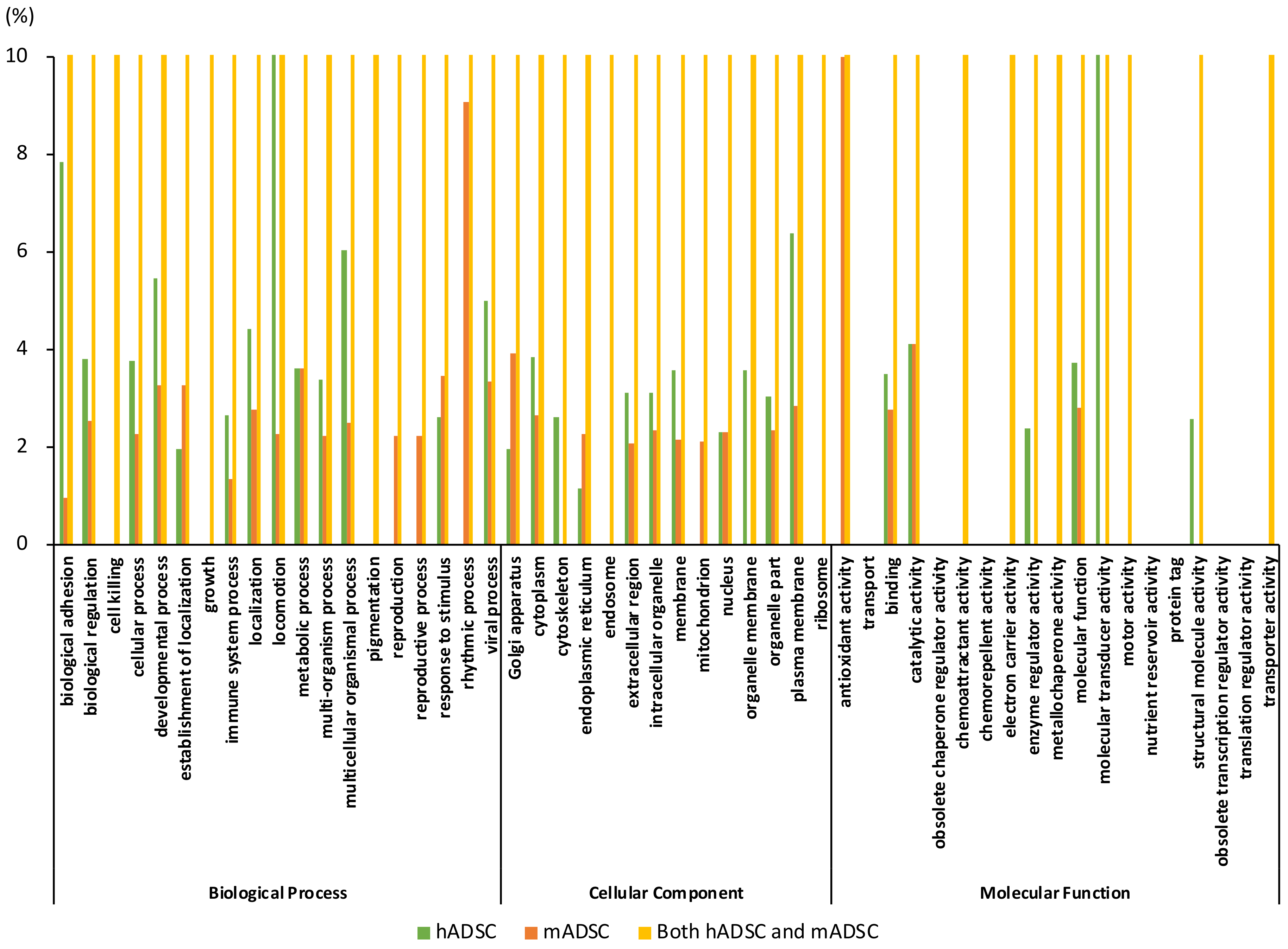

Nahar et al. Supplementary Figure 2 (Figure 4)

Supplementary Table 1. Identification of endogenous proteins contained both hADSC and mADSC.

UniProt/SWISS-			Biological Process		Cellular Component	Molecular Function		emPAI[a]	
PROT ID	Alternate ID	biological adhesion	locomotion	rhythmic process	plasma membrane	antioxidant activity	molecular transducer activity	hMSC_P3_D	mouse_D
Both hADSC and mADSC	Actbl2							70.14	166.14
	VIM(Vim)				plasma membrane			453.06	73.019
	MYH9(Myh9)	platelet aggregation, single organisma	axon guidance, blood vessel endothelial cell migration, leukocyte migration, ne		immunological synapse, integrin complex			373.45	194.72
	MYH10(Myh10)	cell adhesion	axon guidance, neuron migration, substrate-dependent cell migration, cell exte		plasma membrane			51.183	30.689
	FLNA(Flna)	platelet aggregation	wound healing, spreading of cells		plasma membrane			366.81	192.6
	Hsp90aa1		neuron migration		apical plasma membrane, basolateral plasma membrane, brush border membrane, ruffle membrane, sperm plasma r			69.192	128.05
	HSP90B1(Hsp90b1)				plasma membrane			76.775	46.563
	TUBB2A							133.64	98.417
	TUBB6(Tubb6)							80.566	34.922
	HSPA5	cell-cell adhesion	axon guidance		CatSper complex, plasma membrane		virus receptor activity	229.38	144.98
	HSPA1A	cell-cell adhesion					virus receptor activity	20.852	3.1747
	ACTN4(Actn4)	focal adhesion assembly, platelet aggregation			lateral plasma membrane, sarcolemma			213.26	142.86
	ACTN1(Actn1)	focal adhesion assembly, platelet aggregation			plasma membrane			154.5	86.776
	GAPDH(Gapdh)				plasma membrane			117.53	41.271
	Ywhab	cell-cell adhesion						22.748	25.398
	PKM(Pkm)	cell-cell adhesion			plasma membrane			177.24	95.242
	TLN1(Tln1)	cell adhesion, platelet aggregation			ruffle membrane			160.18	84.659
	ENO1(Eno1)	cell-cell adhesion			extrinsic component of plasma membrane, plasma membrane			115.64	109
	CLTCL1				plasma membrane			13.27	13.757
	Cltc				clathrin coat of coated pit			101.42	131.22
	ANXA2(Anxa2)	cell-cell adhesion			basolateral plasma membrane, extrinsic component of plasma membrane, sarcolemma			114.69	112.17
	LDHA	cell-cell adhesion						191.46	47.621
	PLEC(Plec)	cell-cell adhesion			plasma membrane, sarcolemma			122.27	77.252
	ALDOA(Aldoa)	cell-cell adhesion						119.43	58.203
	EEF2(Eef2)	cell-cell adhesion			plasma membrane			80.566	79.368
	P4HB(P4hb)				external side of plasma membrane			99.523	64.553
	DYNC1H1(Dync1h1)							90.992	82.543
	VDAC1(Vdac1)				plasma membrane			38.861	138.63
	AHNAK	cell-cell adhesion			T-tubule, sarcolemma			132.7	9.5242
	FLNC(Flnc)				plasma membrane, sarcolemma			134.59	30.689
	UBA1(Ugdh)							69.192	76.193
	COL1A1(Col1a1)		leukocyte migration					90.992	20.107
	S100A11(S100a11)	cell-cell adhesion						23.696	28.573
	FN1	calcium-independent cell-matrix adhe	glial cell migration, leukocyte migration		apical plasma membrane			103.31	19.048
	IQGAP1(Iqgap1)	cell-cell adhesion			extrinsic component of cytoplasmic side of plasma membrane, lateral plasma membrane, plasma membrane			83.409	45.504
	FLNB(Flnb)	cell-cell adhesion			plasma membrane			59.714	44.446
	PGAM1							32.226	104.77
	VCP							58.766	69.844
	PGK1							50.235	58.203
	ATP5B(Atp5b)		inductive cell migration, negative regulation of cell adhesion involved in substr		plasma membrane			41.705	49.737
	Hspa9							27.487	63.495
	ANXA5(Anxa5)				external side of plasma membrane			66.348	30.689
	LRP1(Lrp1)				integral component of plasma membrane		low-density lipoprotein receptor activ	54.027	60.32
	Pdia3							58.766	48.679
	ATP5A1(Atp5a)				plasma membrane			36.018	41.271
	GSN(Gsn)				plasma membrane			40.757	19.048
	WDR1(Wdr1)		neutrophil migration					51.183	52.912
	ACLY(Acly)				plasma membrane			44.548	40.213
	HSPD1(Hspd1)				plasma membrane			36.018	30.689
	KPNB1(Kpnb1)							32.226	52.912
	TAGLN(Tagln)							66.348	16.932
	MSN(Msn)	leukocyte cell-cell adhesion	leukocyte migration		apical plasma membrane, basolateral plasma membrane, microvillus membrane, plasma membrane			48.34	43.388
	VCL(Vcl)	cell-matrix adhesion, epithelial cell-cell adhesion, platelet aggregation			plasma membrane, sarcolemma			68.244	15.874
	Thbs1	cell adhesion	cell migration		external side of plasma membrane			62.557	21.165
	ASS1(Ass1)							12.322	33.864
	LGALS1							74.879	4.233
	Fasn	cell-cell adhesion			plasma membrane			0.94783	86.776
	ANXA6(Anxa6)						ligand-gated ion channel activity	49.287	25.398
	ARF4(Arf4)		cell migration		ruffle membrane			16.113	13.757
	Vdac2							24.644	41.271
	DPYSL2(Dpysl2)		axon guidance					54.974	37.039
	CAP1(Cap1)		ameboidal-type cell migration		plasma membrane			49.287	28.573
	HSPA4							31.279	45.504
	PLS3(Pls3)							29.383	31.747
	SPTAN1(Sptan1)	cell-cell adhesion	axon guidance					19.905	33.864
	AARS(Aars)							36.966	40.213
	MYOF(Myof)				caveola			48.34	22.223
	P4HA1(P4ha1)							36.018	14.815
	MAP1B				plasma membrane			58.766	6.3495
	Nme2	cell adhesion			ruffle membrane			21.8	43.388
	Nme1				ruffle membrane			7.5827	19.048
	PSMD2							25.592	44.446

[a]Exponentially Modified Protein Abundance Index (http://www.matrixscience.com/help/quant_empai_help.html).

Table 1. Cont 1.

UniProt/SWISS-PROT ID	Alternate ID	Biological Process: biological adhesion	Biological Process: locomotion	Biological Process: rhythmic process	Cellular Component: plasma membrane	Molecular Function: antioxidant activity	Molecular Function: molecular transducer activity	emPAI[a] hMSC_P3_D	emPAI[a] mouse_D
	IPO5(Ipo5)							44.548	27.514
	Myl9	platelet aggregation						39.809	26.456
	MYL12B							31.279	13.757
	PRDX1	cell-cell adhesion	germ cell migration			peroxiredoxin activity, thioredoxin peroxidase activity		30.331	33.864
	CFL1(Cfl1)				lamellipodium membrane, ruffle membrane			40.757	16.932
	EEF1G(Eef1g)	cell-cell adhesion						29.383	24.34
	FSCN1(Fscn1)	cell-cell adhesion	cell migration, cell motility		cell projection membrane			29.383	10.582
	SND1	cell-cell adhesion						36.966	24.34
	CCT6A(CCct6a)							26.539	17.99
	TAGLN2	cell-cell adhesion						40.757	15.874
	CCT5(Cct5)							24.644	34.922
	SPTBN1(Sptbn1)	cell-cell adhesion	axon guidance		axolemma, plasma membrane			16.113	40.213
	PRDX6	cell-cell adhesion				glutathione peroxidase activity, peroxiredoxin activity		25.592	34.922
	CCT2(Cct2)							36.966	24.34
	COPG1							28.435	22.223
	TPI1(Tpi1)							45.496	8.4659
	COL12A1	cell adhesion						48.34	7.4077
	TCP1(Tcp1)							20.852	25.398
	CCT7(Cct7)							32.226	29.631
	Calu							21.8	25.398
	PFN1	cell-cell adhesion						66.348	2.1165
	P4HA2(P4ha2)							32.226	21.165
	Ppia							25.592	25.398
	GSTP1				plasma membrane	glutathione peroxidase activity		54.974	2.1165
	Clic1	platelet aggregation			plasma membrane			27.487	21.165
	Pdia6	platelet aggregation			plasma membrane			25.592	33.864
	Copb1				plasma membrane			21.8	28.573
	Hdlbp	cell-cell adhesion			plasma membrane			29.383	21.165
	CKAP4				plasma membrane			62.557	4.233
	GDI2(Gdi2)							36.018	33.864
	PGD(Pgd)							15.165	16.932
	MDH2(Mdh2)				plasma membrane			24.644	22.223
	PSMD1							24.644	22.223
	UGDH							28.435	13.757
	COPB2							29.383	12.699
	MAPK3	melanocyte adhesion	axon guidance, cell chemotaxis, cell mi	ovarian follicle development	apical plasma membrane, caveola, cytoplasmic side of plasma membrane, exte		VEGF-A-activated receptor activity, V	13.27	26.456
	Mapk1				caveola			8.5305	19.048
	Phgdh							35.07	11.641
	COPA(Copa)							28.435	14.815
	VAT1							27.487	15.874
	HNRNPU(Hnrnpu)			circadian regulation of gene expression				19.905	26.456
	HYOU1(Hyou1)							36.018	8.4659
	PPIB							31.279	6.3495
	COL1A2		leukocyte migration					48.34	4.233
	CNN3(Cnn3)	cell-cell adhesion						35.07	15.874
	Vars							14.218	31.747
	NCL(Ncl)							14.218	7.4077
	SEC31A(Sec31a)							19.905	19.048
	GNAS	platelet aggregation			heterotrimeric G-protein complex, plasma membrane			22.748	13.757
Both hADSC and mADSC	GNAI2				heterotrimeric G-protein complex, plasma membrane			14.218	11.641
	ATP6V1A				apical plasma membrane, integral component of plasma membrane			29.383	9.5242
	Wars							34.122	4.233
	ITGB1	calcium-independent cell-matrix adhe	cell migration involved in sprouting angiogenesis, formation of radial glial scaff		external side of plasma membrane, integrin alpha1-beta1 complex, integrin alph		coreceptor activity, virus receptor ac	24.644	15.874
	RRBP1						receptor activity	33.174	5.2912
	Ugp2							20.852	6.3495
	CTSD							42.653	1.0582
	CNN2	cell-cell adhesion						33.174	13.757
	Capns1				plasma membrane			19.905	20.107
	Rplp0							14.218	24.34
	Nap1l1							13.27	24.34
	Atp2a2				extrinsic component of cytoplasmic side of plasma membrane, integral component of plasma membrane			18.957	17.99
	LDHB							54.974	7.4077
	GFPT1			circadian regulation of gene expression				20.852	17.99
	IPO7				integral component of plasma membrane			16.113	20.107
	FAM129B	cell-cell adhesion			plasma membrane			24.644	12.699
	P3H1(P3h1)				plasma membrane			18.957	14.815
	PDCD6IP(Pdcd6ip)				immunological synapse			18.957	17.99
	TXNRD1					thioredoxin-disulfide reductase activity		27.487	9.5242
	PPP2R1A							17.061	19.048
	CLIC4				plasma membrane			30.331	6.3495
	PDIA4							9.4783	25.398
	RPL9							20.852	17.99
	Psap							0.94783	42.33
	USO1(Uso1)	cell-cell adhesion						19.905	2.1165
	CAPZA1	cell-cell adhesion						18.957	12.699
	DDX3X(Ddx3x)	cell-cell adhesion						10.426	16.932
	ARHGDIA(Arhgdia)				immunological synapse			13.27	23.281
	Rab1b							9.4783	6.3495
	GCN1(Gcn1)	cell-cell adhesion						21.8	10.582
	HNRNPF(Hnrnpf)							14.218	15.874
	HSPB1	platelet aggregation			plasma membrane			40.757	1.0582
	Prep							11.374	24.34
	Rplp2							20.852	11.641
	Sec23a							18.009	9.5242
	SERPINH1(Serpinh1)							69.192	49.737
	CCT8	cell-cell adhesion						16.113	11.641
	PFKP	cell-cell adhesion						33.174	2.1165
	G6PD				cytoplasmic side of plasma membrane			24.644	7.4077
	PALLD		cell migration		plasma membrane			23.696	8.4659
	PTBP1							19.905	13.757
	SARS(Sars)							18.957	14.815
	ARPC2				muscle cell projection membrane, plasma membrane			18.957	11.641
	CAPN2				plasma membrane			36.966	14.815
	ITGAV(Itgav)	cell adhesion mediated by integrin, ce	cell migration, endothelial cell migration, leukocyte migration, negative chemot		alphav-beta3 integrin-IGF-1-IGF1R complex, external side of plasma membrane,		coreceptor activity, virus receptor ac	18.957	9.5242
	MYO1C(Myo1c)				basal plasma membrane, lateral plasma membrane, ruffle membrane, stereocilium membrane			17.061	8.4659
	DDOST(Ddost)							16.113	16.932
	PYGB(Pygb)				plasma membrane			17.061	11.641
	CCT4							20.852	11.641
	Tpm4							9.4783	37.039
	ATP6V1B2				plasma membrane			18.009	12.699

[a]Exponentially Modified Protein Abundance Index (http://www.matrixscience.com/help/quant_empai_help.html).

Nahar et al. Supplementary Table 1 (continuation 1)

Table 1. Cont 2.

UniProt/SWISS-PROT ID	Alternate ID	Biological Process: biological adhesion	Biological Process: locomotion	Biological Process: rhythmic process	Cellular Component: plasma membrane	Molecular Function: antioxidant activity	Molecular Function: molecular transducer activity	emPAI[a] hMSC_P3_D	emPAI[a] mouse_D
	DPYSL3(Dpysl3)							27.487	10.582
	Ppp1ca			circadian regulation of gene expressio	plasma membrane			12.322	17.99
	Tpt1							11.374	21.165
	UCHL1				plasma membrane			21.8	4.233
	Gsto1					glutathione dehydrogenase (ascorbate) activity		1.8957	34.922
	PLOD1(Plod1)							18.957	12.699
	Dstn							8.5305	21.165
	Rars	cell-cell adhesion						15.165	14.815
	CCT3(CCct3)				plasma membrane			15.165	13.757
	Anxa4				apical plasma membrane, plasma membrane			18.009	11.641
	ATL3(Atl3)							21.8	8.4659
	BZW1	cell-cell adhesion						8.5305	21.165
	MVP							39.809	2.1165
	Aldh18a1							8.5305	21.165
	STAT3				plasma membrane			2.8435	25.398
	TARS(Tars)							10.426	12.699
	Ctsb							0.94783	33.864
	CSRP1	platelet aggregation						19.905	5.2912
	GANAB(Ganab)							44.548	11.641
	ARHGAP1	cell-cell adhesion						16.113	9.5242
	Sec22b							14.218	12.699
	STIP1(Stip1)							14.218	12.699
	EPRS							31.279	7.4077
	IARS							37.913	6.3495
	Lars							11.374	12.699
	Fh							14.218	13.757
	PARK7	cell-cell adhesion			plasma membrane	peroxiredoxin activity		21.8	3.1747
	Hspe1							4.7392	23.281
	ASNS							15.165	11.641
	Atp1a1				T-tubule, apical plasma membrane, basolateral plasma membrane, caveola, integral component of plasma membrane			7.5827	17.99
	Cs							1.8957	27.514
	GLG1		leukocyte migration		plasma membrane			15.165	4.233
	NPM1							11.374	15.874
	LRRC59	cell-cell adhesion						17.061	10.582
	CTNNA1	epithelial cell-cell adhesion		ovarian follicle development	catenin complex, flotillin complex, plasma membrane			18.957	10.582
	EHD1	cell-cell adhesion			ciliary pocket membrane			11.374	13.757
	FERMT2	focal adhesion assembly, substrate adhesion-dependent cell spreading			extrinsic component of cytoplasmic side of plasma membrane, lamellipodium membrane			21.8	4.233
	GLS							14.218	6.3495
	SEC13							10.426	7.4077
	TXN							35.07	14.815
	TNC	cell adhesion						31.279	9.5242
	ALDH7A1							11.374	12.699
	DHX9			circadian rhythm				12.322	13.757
	GSPT1(Gspt1)							7.5827	8.4659
	KIF5B(Kif5b)	cell-cell adhesion	axon guidance, motor neuron axon guidance					12.322	8.4659
	MDH1							13.27	4.233
	NOMO1							12.322	12.699
	TPM1				ruffle membrane			39.809	6.3495
	Por							1.8957	22.223
Both hADSC and mADSC	Ptgs1					peroxidase activity		0.94783	24.34
	ANXA1		granulocyte chemotaxis, monocyte che	estrous cycle	apical plasma membrane, basolateral plasma membrane, cornified envelope, extrinsic component of external side of			35.07	3.1747
	COMT				postsynaptic membrane			15.165	7.4077
	EIF3I							12.322	9.5242
	EIF2S1							14.218	6.3495
	Eif4g1	cell-cell adhesion						6.6348	20.107
	PPA1							12.322	8.4659
	LTA4H				plasma membrane			12.322	12.699
	RPL5							12.322	9.5242
	STRAP							12.322	10.582
	PRDX4					peroxidase activity, peroxiredoxin activity, thioredoxin peroxidase activity		23.696	9.5242
	Glud1							1.8957	19.048
	GOT1							13.27	8.4659
	AP2A1				AP-2 adaptor complex, apical plasma membrane, basolateral plasma membrane			21.8	7.4077
	SHMT2							9.4783	10.582
	Me1							0.94783	23.281
	Pcbp2							15.165	19.048
	PSMD3							16.113	8.4659
	Ahcy							7.5827	20.107
	SDHA(Sdha)							8.5305	7.4077
	TXNDC5							19.905	4.233
	UGGT1(Uggt1)							28.435	9.5242
	CNN1							8.5305	14.815
	Dctn1							9.4783	11.641
	EIF3B							8.5305	16.932
	MAPRE1	cell-cell adhesion			cell projection membrane			5.687	15.874
	PAICS(Paics)	cell-cell adhesion						8.5305	5.2912
	Ubr4							7.5827	10.582
	PGM3							14.218	6.3495
	ALDH1L2							18.957	1.0582
	FKBP10							29.383	2.1165
	Pck2							9.4783	11.641
	PSMC3							15.165	6.3495
	Psmd7							7.5827	13.757
	RCN1							11.374	6.3495
	Gdi1							24.644	12.699
	Gpd2							1.8957	15.874
	Ipo4							2.8435	20.107
	Ap1b1							7.5827	14.815
	FKBP9							15.165	6.3495
	NPEPPS				plasma membrane			5.687	14.815
	PSMB1							7.5827	14.815
	PSMB5(Psmb5)							12.322	8.4659
	RTN4	cell-cell adhesion	cerebral cortex radial glia guided migration		plasma membrane			12.322	9.5242
	SET							5.687	16.932
	XPO1(Xpo1)							6.6348	8.4659
	PRMT1							3.7913	16.932
	ARPC1B							17.061	6.3495
	CRTAP							9.4783	9.5242
	EHD2				caveola			15.165	4.233
	EIF3H(Eif3h)							2.8435	11.641

[a]Exponentially Modified Protein Abundance Index (http://www.matrixscience.com/help/quant_empai_help.html).

Table 1. Cont 3.

UniProt/SWISS-PROT ID	Alternate ID	Biological Process: biological adhesion	Biological Process: locomotion	Biological Process: rhythmic process	Cellular Component: plasma membrane	Molecular Function: antioxidant activity	Molecular Function: molecular transducer activity	emPAI[a] hMSC_P3_D	emPAI[a] mouse_D
Both hADSC and mADSC	Gba							0.94783	16.932
	Plod3							5.687	25.398
	Cad							5.687	10.582
	Rcn2							0.94783	22.223
	RPL3							11.374	10.582
	Rpn2							4.7392	22.223
	Flii							3.7913	15.874
	ATIC	cell-cell adhesion						16.113	4.233
	MMP14		astrocyte cell migration, cell migration	ovarian follicle development	integral component of plasma membrane			14.218	5.2912
	Rpl7							7.5827	8.4659
	YARS							14.218	5.2912
	TKT(Tkt)							36.018	32.806
	CAPN1				plasma membrane			15.165	3.1747
	LAMC1	substrate adhesion-dependent cell sp	axon guidance, cell migration					18.009	1.0582
	Sh3bgrl3							1.8957	12.699
	COL6A2	cell adhesion			sarcolemma			16.113	1.0582
	DBN1	cell-cell adhesion			plasma membrane			16.113	1.0582
	EIF3A							12.322	12.699
	IDH2							9.4783	8.4659
	Mrc2				integral component of plasma membrane		transmembrane signaling receptor ac	2.8435	20.107
	VDAC3							10.426	13.757
	SDCBP	cell-cell adhesion	substrate-dependent cell migration, cell extension		interleukin-5 receptor complex			13.27	5.2912
	EIF5B							4.7392	12.699
	MYO1B	cell-cell adhesion			basolateral plasma membrane, lateral plasma membrane, plasma membrane			12.322	1.0582
	Ecm29							5.687	8.4659
	EEF1D	cell-cell adhesion						13.27	6.3495
	EIF4H	cell-cell adhesion						9.4783	6.3495
	NAA15							4.7392	10.582
	PHB2							8.5305	9.5242
	SRM							12.322	3.1747
	Usp14				plasma membrane			5.687	12.699
	Dnajb11							3.7913	9.5242
	FKBP1A							9.4783	7.4077
	RAP1GDS1							10.426	4.233
	Pcna			estrous cycle				4.7392	9.5242
	Akt1				ruffle membrane			4.7392	11.641
	COL5A1	cell adhesion	wound healing, spreading of epidermal cells					15.165	3.1747
	CORO1C		neural crest cell migration		flotillin complex, lateral plasma membrane			11.374	4.233
	Cops7a							4.7392	13.757
	Hnrnpm				integral component of plasma membrane			8.5305	8.4659
	OLA1	cell-cell adhesion						5.687	10.582
	PAK2	cell-cell adhesion			plasma membrane			4.7392	9.5242
	PTPN11		intestinal epithelial cell migration, leukocyte migration					5.687	10.582
	SEC24D							10.426	4.233
	Napa							4.7392	11.641
	Dars							9.4783	3.1747
	ZYX	cell adhesion, cell-matrix adhesion			integral component of plasma membrane			18.957	2.1165
	TGFB1I1	cell adhesion						10.426	5.2912
	Canx							7.5827	24.34
	HNRNPH1							11.374	14.815
	Idh1	cell-cell adhesion						11.374	22.223
	KPNA6(Kpna6)							10.426	4.233
	OTUB1							4.7392	8.4659
	PDLIM1	cell-cell adhesion						8.5305	7.4077
	PSME2							4.7392	8.4659
	Psat1							3.7913	19.048
	Ctnnb1	cell-matrix adhesion, single organisma	layer formation in cerebral cortex, neuron migration		basolateral plasma membrane, catenin complex, flotillin complex, lateral plasma membrane, microvillus membrane, p			9.4783	4.233
	FHL1				plasma membrane			12.322	3.1747
	CTNND1	single organismal cell-cell adhesion			flotillin complex, plasma membrane			9.4783	6.3495
	Tomm40							1.8957	12.699
	Glrx3							2.8435	10.582
	LIMA1	cell-cell adhesion			plasma membrane			9.4783	3.1747
	PICALM	cell-cell adhesion			AP-2 adaptor complex, clathrin coat of coated pit, postsynaptic membrane, presynaptic membrane			10.426	2.1165
	CBX3			rhythmic process				4.7392	10.582
	ARCN1							6.6348	5.2912
	COLGALT1							6.6348	5.2912
	LMAN2				integral component of plasma membrane			7.5827	5.2912
	PRDX5					peroxidase activity, peroxynitrite reductase activity, thioredoxin peroxidase		20.852	1.0582
	HNRNPA3							3.7913	10.582
	Uba2							7.5827	7.4077
	TMED7							10.426	5.2912
	CDC37							8.5305	3.1747
	PSMD14							3.7913	8.4659
	FAM114A1							8.5305	6.3495
	NANS							12.322	1.0582
	PDLIM7							13.27	1.0582
	Pgls							4.7392	9.5242
	ABCE1							3.7913	8.4659
	Eif3k							7.5827	6.3495
	GBE1							9.4783	2.1165
	Lgals3		eosinophil chemotaxis, macrophage chemotaxis, monocyte chemotaxis, monc		external side of plasma membrane, immunological synapse			0.94783	15.874
	NACA							7.5827	7.4077
	PGM1							12.322	2.1165
	Adsl							7.5827	5.2912
	TALDO1							3.7913	10.582
	TAX1BP3				plasma membrane			10.426	4.233
	USP5							3.7913	9.5242
	Ipo9							1.8957	11.641
	AKR1B1							9.4783	2.1165
	FHL2							10.426	1.0582
	CAPG	cell-cell adhesion						15.165	4.233
	Eif3d							6.6348	6.3495
	EIF3F							5.687	7.4077
	LIMS1				plasma membrane			5.687	8.4659
	PSMB6	cell-cell adhesion						5.687	7.4077
	PSMD13							5.687	8.4659
	PSMD4							7.5827	4.233
	CTPS1							5.687	13.757
	RSU1							10.426	3.1747
	KARS				plasma membrane			5.687	7.4077

[a]Exponentially Modified Protein Abundance Index (http://www.matrixscience.com/help/quant_empai_help.html).

Table 1. Cont 4.

UniProt/SWISS-PROT ID	Alternate ID	Biological Process: biological adhesion	Biological Process: locomotion	Biological Process: rhythmic process	Cellular Component: plasma membrane	Molecular Function: antioxidant activity	Molecular Function: molecular transducer activity	emPAI[a]: hMSC_P3_D	emPAI[a]: mouse_D
Both hADSC and mADSC	Akr1a1				apical plasma membrane			7.5827	4.233
	CNPY2				integral component of plasma membrane			6.6348	5.2912
	AP2A2				AP-2 adaptor complex, plasma membrane			8.5305	6.3495
	Ero1a							5.687	6.3495
	Nars							6.6348	14.815
	Sec23ip							3.7913	9.5242
	ERP29							12.322	4.233
	GPI				ciliary membrane, plasma membrane			17.061	4.233
	ITGA5	cell adhesion mediated by integrin, ce	leukocyte migration, wound healing, spreading of epidermal cells		alphav-beta3 integrin-vitronectin complex, external side of plasma membrane, i		virus receptor activity	10.426	1.0582
	LASP1	cell-cell adhesion						10.426	1.0582
	MIF		leukocyte migration, positive chemotaxis					13.27	3.1747
	CYB5R3							17.061	1.0582
	Hnrnpab							3.7913	11.641
	HARS							9.4783	2.1165
	Cpne1				plasma membrane			4.7392	7.4077
	ESD							13.27	15.874
	OAT							13.27	5.2912
	Kpna3							9.4783	9.5242
	ANXA11							9.4783	1.0582
	C1qbp				plasma membrane			0.94783	14.815
	RCN3							10.426	1.0582
	Eef1b							8.5305	25.398
	CARS							14.218	3.1747
	KPNA4							6.6348	11.641
	ESYT1(Esyt1)				plasma membrane			17.061	1.0582
	Pdlim5	cell-cell adhesion			plasma membrane, postsynaptic membrane			6.6348	9.5242
	UAP1				plasma membrane			6.6348	5.2912
	LAP3							12.322	5.2912
	ADH5							14.218	2.1165

[a]Exponentially Modified Protein Abundance Index (http://www.matrixscience.com/help/quant_empai_help.html).
